# Determinants of Elevated Blood Pressure and Hypertension Among Undergraduate Medical Students in Central Karnataka, India: A Cross-Sectional Study

**DOI:** 10.7759/cureus.103132

**Published:** 2026-02-06

**Authors:** Shalini H, Kashavva B Andanigoudar, Vidya GS, Sharath Chandra H

**Affiliations:** 1 Community Medicine, Jagadguru Jayadeva Murugarajendra (JJM) Medical College, Davangere, IND; 2 Community Medicine, Jagadguru Gangadhar Mahaswamigalu Moorsavirmath Medical College, Karnataka Lingayat Education (KLE) Academy of Higher Education and Research, Hubballi, IND; 3 Pediatric Dentistry, SJM Dental College, Chitradurga, IND

**Keywords:** blood pressure, body mass index, hypertension, medical students, prehypertension, waist circumference

## Abstract

Background

Non-communicable diseases (NCDs), including cardiovascular diseases, cancers, chronic respiratory diseases, and diabetes, are the leading causes of mortality worldwide. Elevated blood pressure levels are an important precursor to hypertension and cardiovascular disease, particularly among young adults, yet their determinants remain underexplored in this population.

Objectives

The objective of this study is to assess the prevalence of elevated blood pressure levels, hypertension, and its associated determinants among undergraduate medical students.

Methods

A cross-sectional study was conducted among undergraduate students of Jagadguru Jayadeva Murugarajendra (JJM) Medical College, Davangere, India, from August 2024 to October 2024. Students with a known diagnosis of hypertension or those currently receiving antihypertensive treatment were excluded. The estimated sample size was 220. A total of 220 students participated in the study and completed the study protocol. Data were collected using a pre-tested, semi-structured questionnaire, along with anthropometric measurements and blood pressure recordings, after obtaining informed written consent. Associations between blood pressure status and selected determinants were analyzed.

Results

Of the 220 participants, 119 (54%) were male and 101 (45.9%) were female. The majority of students were 21 years old (52.7%). A family history of hypertension was reported by 72 participants (32.7%), and 63 (28.5%) were classified as obese based on BMI. Based on blood pressure measurements, elevated blood pressure levels were observed in 67 (30.45%) participants, and 56 (25.45%) were found to have Stage I hypertension, while 20 (9.09%) were classified as having Stage II hypertension. BMI, gender, dietary patterns, and waist circumference showed a statistically significant association with blood pressure levels.

Conclusion

The study highlights a substantial burden of elevated blood pressure levels and hypertension among undergraduate medical students. Modifiable risk factors such as increased BMI, dietary patterns, and waist circumference were significantly associated with elevated blood pressure, emphasizing the need for early screening and lifestyle-based interventions among young adults.

## Introduction

Non-communicable diseases (NCDs), including cardiovascular diseases, cancers, chronic respiratory diseases, and diabetes, remain the leading cause of global mortality, accounting for nearly 74% of all deaths worldwide. A disproportionate burden is borne by low- and middle-income countries, where over 75% of NCD-related deaths and approximately 86% of the 17 million premature deaths (occurring before 70 years of age) are reported annually. This epidemiological shift underscores the urgent need for early identification and control of cardiovascular risk factors, particularly among younger populations [1].

Hypertension is a major modifiable risk factor for NCDs and a leading contributor to cardiovascular morbidity and mortality. According to recent World Health Organization estimates, approximately 1.28 billion adults aged 30-79 years worldwide are living with hypertension, with nearly two-thirds residing in low- and middle-income countries. In India, the challenge is amplified by its demographic profile, where nearly 80% of the population falls within the economically productive age group of 15-64 years. Alarmingly, about one-fifth of individuals aged 30 years are projected to die prematurely due to NCDs, highlighting the long-term implications of uncontrolled cardiovascular risk factors [2].

Contemporary hypertension guidelines have emphasized the importance of early blood pressure abnormalities. The American College of Cardiology/American Heart Association (ACC/AHA) 2017 guidelines reclassified blood pressure categories, introducing the term elevated blood pressure for systolic blood pressure values of 120-129 mmHg with diastolic blood pressure <80 mmHg, and defining Stage I hypertension as blood pressure levels of 130-139/80-89 mmHg [3]. Evidence suggests that individuals within these ranges carry a significantly higher risk of cardiovascular events compared to those with optimal blood pressure, challenging earlier notions that such levels were clinically benign [4].

Although hypertension is formally diagnosed at blood pressure levels ≥140/90 mmHg, accumulating evidence indicates that cardiovascular risk increases in a continuous, graded manner beginning at much lower blood pressure thresholds. Individuals with systolic blood pressure levels in the range of 130-139 mmHg demonstrate nearly twice the risk of coronary and cerebrovascular events compared to those with systolic blood pressure around 115 mmHg [5]. Despite this, early-stage hypertension and elevated blood pressure often remain undetected, particularly among young adults who are perceived to be at low risk [6].

Medical students represent a unique and vulnerable population due to academic stress, irregular lifestyles, physical inactivity, and dietary imbalances. These factors, combined with limited health-seeking behavior, may contribute to the under-recognition of elevated blood pressure and hypertension in this group. As future healthcare providers, the cardiovascular health of medical students assumes added significance.

Therefore, the present study was conducted to assess the prevalence and determinants of elevated blood pressure and hypertension among undergraduate medical students, with the aim of facilitating early identification and promoting preventive strategies in this high-risk yet often overlooked population.

## Materials and methods

Study design and participants

**Figure 1:**
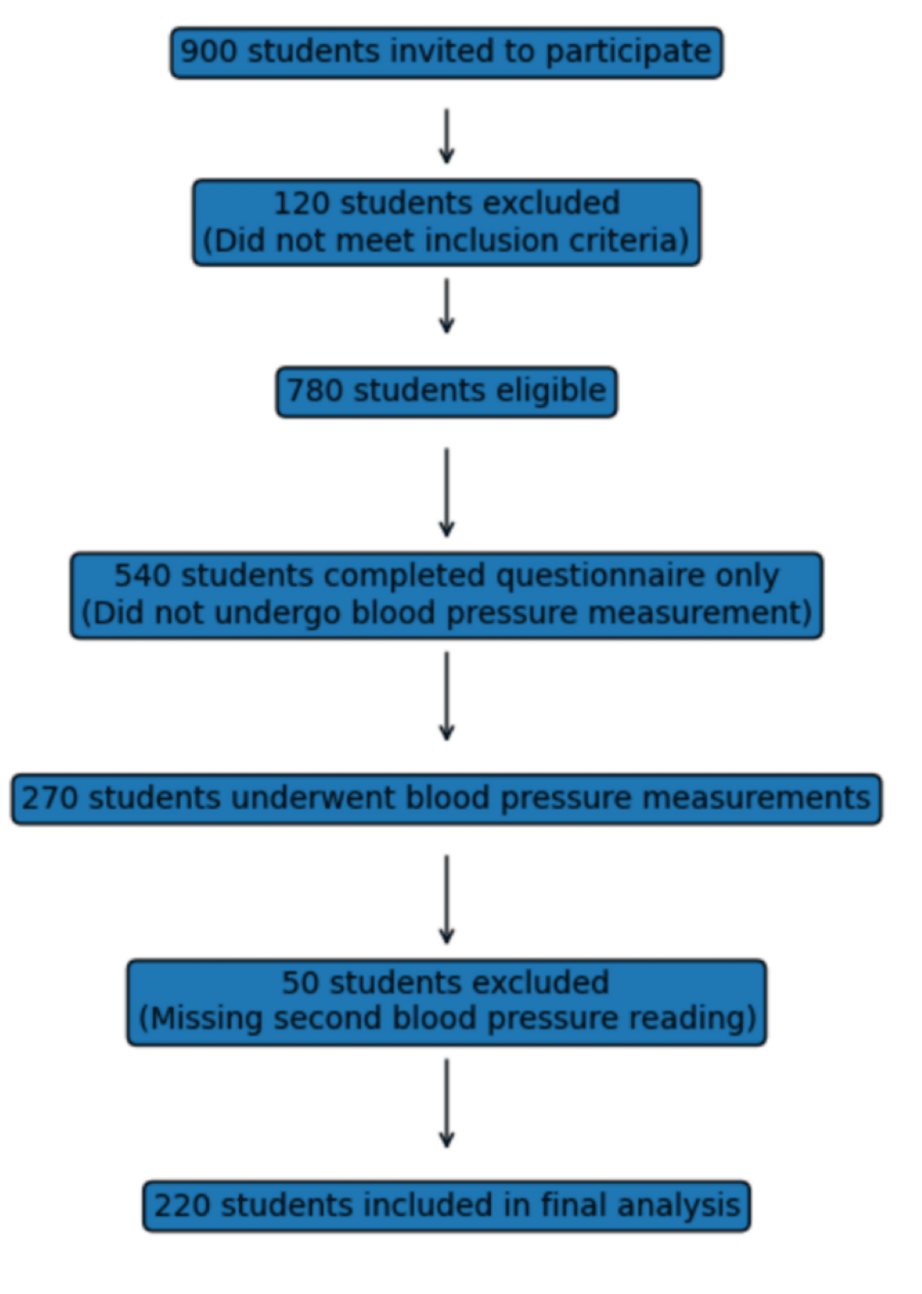
Flow chart showing the selection of study participants

A cross-sectional study was conducted among undergraduate medical students of Jagadguru Jayadeva Murugarajendra (JJM) Medical College, Davangere, India, from August 2024 to October 2024. All undergraduate students were invited to participate in the study. Students with a known diagnosis of hypertension, those currently on antihypertensive treatment, individuals receiving medications known to affect blood pressure (such as corticosteroids), and those who did not provide informed consent were excluded. Only students who voluntarily consented and were willing to participate were enrolled in the study. Considering a hypertension prevalence of 16.3%, with a 5% margin of error and a 95% confidence level, the estimated sample size was 209. After accounting for a 5% non-response rate, the final sample size was increased to 220 [7]. The flow chart for the selection of study participants is provided in Figure 1.

Ethical considerations

Ethical approval was obtained from the Institutional Ethics Committee of JJM Medical College prior to the commencement of the study. Written informed consent was obtained from all participants before data collection.

Data collection tool

Data were collected using a pre-tested, semi-structured questionnaire administered by trained personnel (see Appendix A). The questionnaire was validated through pilot testing on 10 undergraduate students and reviewed by subject experts for content validity. It comprised two sections: (i) socio-demographic characteristics, family history of hypertension, lifestyle factors, and habits; (ii) anthropometric measurements, including height, weight, waist circumference, and blood pressure.

Anthropometric Measurements

Body weight was measured to the nearest 0.1 kg using a calibrated digital weighing scale, with participants wearing light clothing. Height was measured to the nearest 0.5 cm using a stadiometer, with participants standing barefoot in an upright position with heels, buttocks, and occiput touching the wall. BMI was calculated as weight (kg)/height² (m²) and classified according to the WHO adult BMI classification as follows: normal (18.5-22.9 kg/m²), overweight (23.0-24.9 kg/m²), obese class I (25.0-29.9 kg/m²), and obese class II (≥30 kg/m²) [8].

Waist circumference was measured using a non-stretchable measuring tape placed horizontally at the level of the iliac crest, at the end of normal expiration, ensuring that the tape was snug but did not compress the skin, and was parallel to the floor. Measurements were recorded in centimeters. Waist circumference cut-offs were categorized as low, high, and very high risk based on standard gender-specific criteria.

The cut-offs for men and women were adopted from a previous study [9]. In men, the low-risk category implies a waist circumference of less than 94 cm, high risk implies a waist circumference in the range of 94 to 102 cm, and very high risk implies a waist circumference greater than 102 cm. In women, the low-risk category implies a waist circumference of less than 80 cm, high risk implies a waist circumference in the range of 80 to 88 cm, and very high risk implies a waist circumference greater than 88 cm. 

Blood pressure measurement: Blood pressure was measured using a standard mercury sphygmomanometer on two separate occasions, with a minimum interval of 10 minutes between readings. Participants were seated comfortably in a 90-degree position during the measurement. The average of the two readings was used for analysis.

Blood pressure categories were defined according to current guidelines: elevated blood pressure was defined as systolic blood pressure of 120-129 mmHg with diastolic blood pressure <80 mmHg; Stage I hypertension as systolic blood pressure of 130-139 mmHg or diastolic blood pressure of 80-89 mmHg; and Stage II hypertension as systolic blood pressure ≥140 mmHg or diastolic blood pressure ≥90 mmHg [3].

Statistical analysis

Data were entered into Microsoft Excel (Microsoft® Corp., Redmond, WA, USA) and analyzed using IBM SPSS Statistics for Windows, Version 20 (Released 2011; IBM Corp., Armonk, NY, USA). Descriptive statistics were expressed as frequencies and percentages. The Chi-square test was applied to assess the association between blood pressure status and selected socio-demographic and anthropometric variables. A p-value of <0.05 was considered statistically significant.

## Results

A total of 220 participants were included in the study, out of which 119 (54.09%) were male and 101 (45.9%) were female. The largest age group was 21 years old, making up 52.7% (116) of participants, followed by those aged 22 at 17.2%. Most participants, 125 (56%), lived in hostels. About 40 (38.2%) reported having at least one habit, and 139 (62.7%) reported following a mixed diet. Additionally, 72 (32.7%) had a family history of hypertension (Table 1). About 109 participants (49.5%) had a normal BMI, while 63 (28.5%) were classified as obese based on their BMI measurements (Table 2).

**Table 1:** Demographic profile of the study participants (n = 220)

Variable	n (%)
Gender	
Female	101 (45.9)
Male	119 (54)
Age (in years)	
20	17 (7.7)
21	116 (52.7)
22	71 (32.3)
23	13 (5.9)
24	3 (1.4)
Place of stay	
Home	74 (33.6)
Hostel	125 (56.8)
PG	21 (9.5)
Habits	
Alcohol	28 (12.7)
Smoking	12 (5.5)
None	180 (81.8)
Diet	
Mixed	139 (62.7)
Vegeterian	81 (36.8)
Family history of hypertension	
No	148 (67.3)
Yes	72 (32.7)

**Table 2:** Distribution of study participants based on body mass index

BMI (kg/m^2^)	n (%)	Mean with SD
Normal (18.5-22.9)	109 (49.5)	23 ± 4.07
Overweight (23-24.9)	48 (21.5)	
Obese (≥25)	63 (28.5)	

Based on the distribution of participants by waist circumference and associated risk levels for both males and females, among males, 86 (39%) were classified as low risk, with a waist circumference under 94 cm; 21 (9.54%) as high risk (94-102 cm); and 12 (5.45%) as very high risk (over 102 cm). For females, 65 (29.5%) fell into the low-risk group (waist circumference less than 80 cm), 25 (11.36%) were high risk (80-88 cm), and 11 (5%) were very high risk (over 88 cm) (Table 3).

**Table 3:** Distribution of the study participants based on the waist circumference

Waist circumference (in cm) risk categories for men	Male n (%)	Waist circumference (in cm) risk categories for women	Female n (%)
Low risk (<94)	86 (39%)	Low risk (<80)	65 (29.5%)
High risk (94-102)	21 (9.54%)	High risk (80-88)	25 (11.36%)
Very high risk (>102)	12 (5.45%)	Very high risk (>88)	11 (5%)

Among the 220 undergraduate medical students included in the study, the majority, 77 participants (35%), had normal blood pressure (<120/80 mmHg). The mean systolic blood pressure in this group was 120.7 ± 16.2 mmHg, while the mean diastolic blood pressure was 78.1 ± 8.4 mmHg. Elevated blood pressure (systolic blood pressure 120-129 mmHg with diastolic blood pressure <80 mmHg) was observed in 67 participants (30.45%), indicating a substantial proportion of students with blood pressure levels above the optimal range but not meeting criteria for hypertension. Stage I hypertension (systolic blood pressure 130-139 mmHg or diastolic blood pressure 80-89 mmHg) was identified in 56 participants (25.45%), suggesting the study population had clinically significant hypertension requiring lifestyle modification and close monitoring. Stage II hypertension (systolic blood pressure ≥140 mmHg or diastolic blood pressure ≥90 mmHg) was present in 20 (9.09%) participants, reflecting a concerning prevalence of established hypertension among young adults (Table 4).

**Table 4:** Distribution of study participants based on their blood pressure levels SBP, systolic blood pressure; DBP, diastolic blood pressure

Blood pressure (mm of Hg)	n (%)	Mean systolic blood pressure with SD	Mean diastolic blood pressure with SD
Normal (<120/80 mmHg)	77 (35.00%)	120.7 ± 16.2	78.1 ± 8.4
Elevated blood pressure (SBP 120-129 mmHg with DBP <80 mmHg)	67 (30.45%)	-	-
Hypertension stage I (SBP 130-139 mmHg or DBP 80-89 mmHg)	56 (25.45%)	-	-
Hypertension stage II (SBP ≥140 mmHg or DBP ≥90 mmHg)	20 (9.09%)	-	-

Table 5 depicts the various factors and their association with blood pressure levels. BMI was significantly associated with blood pressure categories (χ² = 18.31, p = 0.0011). The proportion of obese participants was highest among those with hypertension (15.45%) compared to those with elevated blood pressure (8.18%) and normal blood pressure (5%).

**Table 5:** Association of risk factors with blood pressure levels (n = 220)

Variable	Normal blood pressure n(%)	Elevated blood pressure n (%)	Hypertension n (%)	p-value	χ²
BMI					
Normal	48 (21.8)	32 (14.5)	29 (13.18)	0.0011	18.31
Overweight	18 (8.18)	17 (7.72)	13 (5.9)		
Obese	11 (5)	18 (8.18)	34 (15.45)		
Gender					
Female	63 (28.6)	29 (13.18)	9 (4.09)	0.001	75.69
Male	14 (6.36)	38 (17.2)	67 (30.45)		
Habits					
Alcohol	15 (6.81)	5 (2.27)	2 (0.9)	0.0069	14.14
Smoking	2 (0.9)	4 (1.81)	6 (2.72)		
None	60 (27.2)	58 (26.36)	68 (30.9)		
Diet					
Mixed	40 (18.18)	42 (19.09)	32 (14.5)	0.049	6.04
Veg	37 (16.8)	25 (11.3)	44 (20)		
Waist circumference					
Low risk (<94 & 80)	71 (32.7)	46 (20.9)	34 (15.45)	<0.001	44.12
High risk (94-102 & 80-88)	3 (1.36)	11 (5)	31 (14.09)		
Very high risk (102 & 88)	3 (1.36)	10 (4.54)	11 (5)		

A highly significant association was observed between gender and blood pressure status (χ² = 75.69, p = 0.001). Males constituted a larger proportion of the hypertensive group (30.45%), whereas females were predominantly represented in the normal blood pressure category (28.6%).

Lifestyle habits, including alcohol consumption and smoking, were significantly associated with blood pressure categories (χ² = 14.14, p = 0.0069). Participants reporting smoking showed a higher proportion in the hypertensive group (2.72%) compared to those with normal blood pressure (0.9%). The majority of participants without these habits were observed in the normal and elevated blood pressure groups.

Dietary pattern demonstrated a statistically significant association with blood pressure status (χ² = 6.04, p = 0.049). A higher proportion of participants consuming a mixed diet was observed among those with elevated blood pressure and hypertension, while a vegetarian diet was more common among normotensive individuals.

Waist circumference showed a strong and statistically significant association with blood pressure categories (χ² = 44.12, p < 0.001). Participants with high-risk and very high-risk waist circumference were predominantly hypertensive (14.09% and 5%, respectively), whereas those with low-risk waist circumference were mostly normotensive (32.7%).

Table 6 shows the multivariate logistic regression of risk factors of hypertension.

**Table 6:** Multivariate logistic regression of the risk factors of hypertension

Variable	Category	Adjusted odds ratio (AOR)	95% confidence interval	p-value
BMI	Normal	1 (Reference)	1.31-5.47	0.006
	Obese	2.68		
Gender	Female	1 (Reference)	5.01-26.01	<0.001
	Male	11.42		
Habits	None	1 (Reference)	0.29-1.71	0.45
	Alcohol/Smoking	0.71		
Diet	Vegetarian	1 (Reference)	0.34-0.99	0.047
	Mixed	0.58		
Waist circumference	Low risk	1 (Reference)	2.45-9.86	<0.001
	High/Very high risk	4.92		

BMI showed a significant association with hypertension. Obese individuals had 2.68 times higher odds of having hypertension compared to those with normal BMI (AOR = 2.68; 95% CI: 1.31-5.47; p = 0.006), indicating obesity as an independent risk factor.

Gender emerged as a strong predictor of hypertension. Males had 11.42 times higher odds of being hypertensive compared to females (AOR = 11.42; 95% CI: 5.01-26.01; p < 0.001). This represents the strongest association observed in the model.

Lifestyle habits (alcohol consumption and smoking) were not significantly associated with hypertension after adjustment. Participants with alcohol or smoking habits had lower odds of hypertension compared to those without habits; however, this association was not statistically significant (AOR = 0.71; 95% CI: 0.29-1.71; p = 0.45).

The dietary pattern showed a statistically significant association. Participants consuming a mixed diet had 42% lower odds of hypertension compared to those on a vegetarian diet (AOR = 0.58; 95% CI: 0.34-0.99; p = 0.047), suggesting a modest protective effect.

Waist circumference demonstrated a strong independent association with hypertension. Individuals with high or very high-risk waist circumference had nearly five times higher odds of hypertension compared to those with low-risk waist circumference (AOR = 4.92; 95% CI: 2.45-9.86; p < 0.001), highlighting central obesity as a major determinant.

Male gender, general obesity, and central obesity were independently associated with hypertension, while waist circumference showed a stronger association than BMI; dietary pattern showed a modest protective effect, whereas lifestyle habits were not significant after adjustment.

## Discussion

The present study was carried out to study the prevalence of elevated blood pressure, hypertension, and associated risk factors among 220 participants, mainly young adults aged 20-24 years. The findings revealed that 34.5% were hypertensive and nearly 30.5% had elevated blood pressure levels, with higher prevalence among males and those with increased BMI and waist circumference. These findings are consistent with trends observed in other studies conducted among medical students and young adults, although with variations in prevalence rates and associated factors.

In a study by Singh et al. in Uttar Pradesh, the prevalence of hypertension was 21.33%, lower than in our study (34.5%). However, their population had a higher proportion of overweight and obese students (60% overweight, 22.67% obese), which may account for the elevated hypertension prevalence [10]. Similarly, Lahole et al. reported a hypertension prevalence of 9.8% and a significant positive correlation between BMI, waist circumference, and blood pressure levels, supporting our finding that obesity and central adiposity are strong predictors of elevated blood pressure [11].

The current study also observed a statistically significant association (p < 0.001) between increased waist circumference and hypertension, which aligns with multiple studies, including the study from Odisha by Patnaik and Choudhury, where 67% of the students were prehypertensive or hypertensive, and waist circumference and BMI were linked to elevated blood pressure [12]. Our results further reinforce the need to monitor central obesity, especially in youth, as a marker for cardiovascular risk.

In contrast, the study by Namita and Ranjan involving 222 medical students found no cases of hypertension, with 26.1% being prehypertensive and only one participant reporting alcohol consumption, which may partly explain their lower hypertension prevalence [13]. Our study showed moderate levels of alcohol use (12.7%) and smoking (5.5%), but these were not statistically significant, suggesting lifestyle variations among study populations.

The prevalence of elevated blood pressure (30.5%) in the current study is notably high. This can be attributed to the level of academic stress, staying away from home (homesickness), and the dietary patterns in the hostels. This is comparable to the 64% prehypertension prevalence reported in the Kolkata study by Chattopadhyay et al., which also highlighted associations with BMI, diet, and family history [14]. These findings indicate that medical students, despite being part of the healthcare system, are not immune to developing early cardiovascular risks.

Gender disparities were significant in blood pressure levels, with males more likely to have elevated blood pressure or hypertension. This aligns with results from Qassim University, where male students had higher prevalence rates of elevated blood pressure and stronger associations with BMI and family history [15].

Globally, the Indonesian adolescent study by Sudikno et al. also reinforces the concern of emerging hypertension in youth, citing obesity and male gender as dominant risk factors, consistent with our findings [16].

In a study done by Thamizhmaran et al., it was observed that the prevalence of prehypertension was 13%, which is lower than the findings in the present study [17]. It was observed that among the study population, gender, family history of hypertension, type of diet, and BMI were significantly associated with the occurrence of prehypertension, which is in line with the present study, where gender, diet, and BMI were significantly associated with the occurrence of elevated blood pressure and hypertension.

In a study carried out by Sharma et al., it was observed that one‑third of the participants (34.5%) were in the pre‑hypertensive stage, and an alarming number of them had an unacceptable BMI (24.7%) and waist‑hip ratio (28.5%), which is consistent with the findings in the present study, where elevated blood pressure was about 30.5% [18].

Limitations

The study population consisted primarily of young adults aged 20-24 years from a single medical college, which may limit the generalizability of the findings to other age groups, institutions, or community settings. Blood pressure was measured during a single visit using the average of two readings, which may have led to misclassification due to transient factors such as stress, anxiety, or recent physical activity.

Lifestyle variables, including dietary habits, alcohol consumption, and smoking, were self-reported and therefore subject to recall bias and social desirability bias, potentially underestimating their true prevalence and association with blood pressure. Additionally, detailed dietary intake and physical activity levels were not quantified, limiting a more nuanced assessment of lifestyle factors. The cross-sectional design of the study also precludes establishing a causal relationship between the identified risk factors and hypertension.

Recommendations

Routine blood pressure screening should be incorporated into periodic health check-ups for medical students to enable early identification of elevated blood pressure and hypertension. Anthropometric assessments, particularly waist circumference measurement, should be routinely used alongside BMI to identify students at higher cardiovascular risk. Lifestyle modification programs, focusing on weight management, regular physical activity, healthy dietary practices, and stress reduction, should be implemented at the institutional level. Hostel-based interventions, including improved dietary quality, reduced salt intake, and promotion of physical activity, should be prioritized, given the high proportion of hostel residents. Health education and counseling sessions, addressing early cardiovascular risk - especially targeting male students - should be conducted regularly.

## Conclusions

The present study demonstrates a high prevalence of elevated blood pressure and hypertension among undergraduate medical students, with nearly two-thirds of participants exhibiting blood pressure levels above the normal range. Although only 35% of students were normotensive, a substantial proportion had elevated blood pressure or Stage I hypertension, indicating early cardiovascular risk in a young population. Male gender, general obesity, and central obesity emerged as independent and significant predictors of hypertension, with waist circumference showing a stronger association than BMI. These findings emphasize the importance of central adiposity as a key determinant of hypertension in young adults. Dietary pattern showed a modest protective association, while lifestyle habits, such as alcohol consumption and smoking, did not demonstrate an independent effect after adjustment. Findings highlight that medical students are vulnerable to early-onset hypertension, underscoring the need for targeted preventive strategies focusing on obesity reduction, regular screening, and lifestyle modification within medical institutions.

## Appendices

Appendix A: Questionnaire

1. Roll no-

2. Sex

3. Age

4. Year of study

5. Place of stay

6. Habits

7. Diet

8. Lifestyle

9. Family history of Hypertension

10. Any history of long-term drug intake

11. If yes, name of the drug

12. Height

13. Weight

14. Waist circumference

15. Body mass index

16. Blood pressure reading 1st

17. Blood pressure reading 2nd

## References

[1] World Health Organization. (2022 (2025). Noncommunicable diseases. https://www.who.int/news-room/fact-sheets/detail/noncommunicable-diseases.

[2] (2025). Hypertension. https://www.who.int/news-room/fact-sheets/detail/hypertension.

[3] Chobanian AV, Bakris GL, Black HR (2003). Seventh report of the Joint National Committee on prevention, detection, evaluation, and treatment of high blood pressure. Hypertension.

[4] Whelton PK, Carey RM, Aronow WS (2017). ACC/AHA/AAPA/ABC/ACPM/AGS/APhA/ASH/ASPC/NMA/PCNA guideline for the prevention, detection, evaluation, and management of high blood pressure in adults. J Am Coll Cardiol.

[5] Vasan RS, Larson MG, Leip EP, Evans JC, O'Donnell CJ, Kannel WB, Levy D (2001). Impact of high-normal blood pressure on the risk of cardiovascular disease. N Engl J Med.

[6] Lewington S, Clarke R, Qizilbash N (2002). Age-specific relevance of usual blood pressure to vascular mortality: a meta-analysis of individual data for one million adults in 61 prospective studies. Lancet.

[7] Thomas A, Singh SP, Pachauri R, Dikshit A (2025). Prevalence of hypertension and its associated risk factors among 30-60 years population in urban and rural area of District Jalaun. Eur J Cardiovasc Med.

[8] Expert Panel on the Identification, Evaluation Evaluation, and Treatment of Overweight and Obesity in Adults (1998). Executive summary of the clinical guidelines on the identification, evaluation, and treatment of overweight and obesity in adults. Arch Intern Med.

[9] National Collaborating Centre for Primary Care and the Centre for Public Health Excellence at NICE (2006). The Prevention, Identification, Assessment and Management of Overweight and Obesity in Adults and Children. Obesity Guidance on the Prevention, Identification, Assessment and Management of Overweight and Obesity in Adults and Children.

[10] Singh AP, Mishra S, Yadav AK, Singh A (2013). The prevalence of hypertension and its modifiable risk factors among medical students of a medical college in Uttar Pradesh, India. J Lumbini Med Coll.

[11] Lahole S, Choudhary RC, Khapre MP, Mudey A (2018). Prevalence of hypertension and its association with anthropometric measurements among medical students. Int J Community Med Public Health.

[12] Patnaik A, Choudhury KC (2015). Assessment of risk factors associated with hypertension among undergraduate medical students in a medical college in Odisha. Adv Biomed Res.

[13] Namita R, Ranjan DP (2017). Study of blood pressure, pre-hypertension, and hypertension in medical students. Natl J Physiol Pharm Pharmacol.

[14] Chattopadhyay A, Taraphdar P, Sahu BK (2014). A study on prevalence of hypertension and its related risk factors
among undergraduate medical students in Kolkata. IOSR J Dent Med Sci.

[15] Al-Majed HT, Sadek AA (2012). Pre-hypertension and hypertension in college students in Kuwait: a neglected issue. J Family Community Med.

[16] Sudikno A, Mubasyiroh R, Rachmalina R, Arfines PP, Puspita T (2023). Prevalence and associated factors for prehypertension and hypertension among Indonesian adolescents: a cross-sectional community survey. BMJ Open.

[17] Thamizhmaran S, Dsouza MJ, Ramadass D, Daniel JA (2024). Rural health dynamics: exploring the prevalence of prediabetes and prehypertension among the rural population of Puducherry district. J Family Med Prim Care.

[18] Sharma SK, Mudgal SK, Thakur K, Gaur R, Aggarwal P (2020). Lifestyle behavior of budding health care professionals: a cross-sectional descriptive study. J Family Med Prim Care.

